# SOD1 amyotrophic lateral sclerosis associated with Neurosarcoidosis: a case report and review of the literature

**DOI:** 10.1093/omcr/omag078

**Published:** 2026-05-24

**Authors:** Al-Alya AlSabah, Haatem Reda

**Affiliations:** Department of Neurology, Massachusetts General Hospital, 55 Fruit Street, Boston, MA 02114, United States; Department of Neurology, Kuwait University, Kuwait; Department of Neurology, Massachusetts General Hospital, 55 Fruit Street, Boston, MA 02114, United States

**Keywords:** amyotrophic lateral sclerosis, motor neuron disease, SOD1, Neurosarcoidosis, neuroinflammation

## Abstract

We describe a 37-year-old man with coexisting amyotrophic lateral sclerosis (ALS) caused by a mutation in superoxide dismutase 1 (SOD1) and probable neurosarcoid myeloradiculitis. The concurrence of the two rare conditions posed significant diagnostic and therapeutic challenges. We discuss the diagnostic timeline, therapeutic interventions, outcomes over half a decade of care, and a review of relevant literature.

## Introduction

Amyotrophic lateral sclerosis (ALS) is a progressive neurodegenerative disease. Mutations in the superoxide dismutase 1 (*SOD1*) gene account for approximately 2% of all ALS cases and typically cause a toxic gain of function through misfolding and aggregation of mutant SOD1 protein. This leads to multiple downstream pathogenic mechanisms, including impaired proteostasis, mitochondrial dysfunction, neuroinflammation, and oxidative stress, ultimately resulting in motor neuron degeneration [1]. In contrast, sarcoidosis is a systemic inflammatory disease characterized by forming granulomas in various organs, including the nervous system. The exact antigens triggering sarcoidosis remain unknown [2]. While the link between these two rare diseases is unclear, we aim to present the first documented case of their coexistence, which posed significant treatment challenges.

## Case report

**Figure 1:**
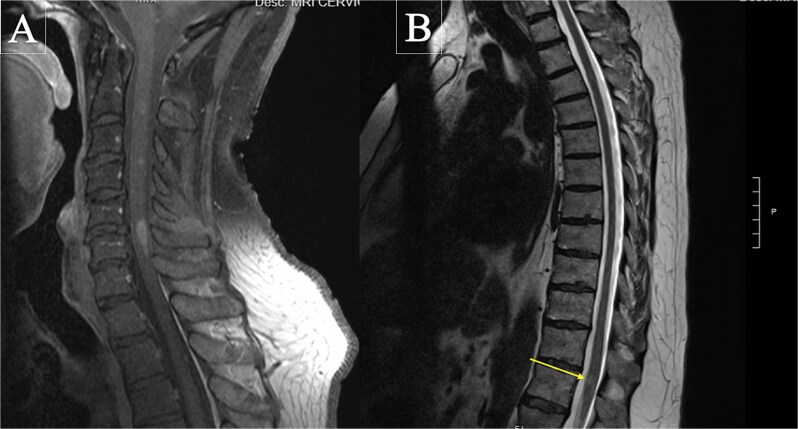
Sagittal T2-weighted magnetic resonance imaging of the spine. (A) Cervical spine demonstrating multiple intramedullary T2-hyperintense lesions. (B) Thoracic spine showing short-segment intramedullary T2-hyperintense lesions (arrow).

A 37-year-old North African man with diabetes and consanguineous parents presented with a five-year history of progressive lower limb weakness, with frequent falls in the final year. He reported no sensory symptoms, bulbar involvement, or systemic complaints. The patient stated that his father had a similar progressive motor syndrome beginning in mid-life and died at age 53; however, formal diagnostic confirmation was not available. Neurological examination revealed thenar and calf wasting without fasciculations, preserved cranial nerve function, and no sensory deficits. Strength was reduced in the distal upper limbs and diffusely reduced in the lower limbs. Reflexes were brisk in the upper limbs and absent in the lower limbs, with extensor plantar responses. Gait was impaired by bilateral foot drop and hip flexor weakness. Electrodiagnostic studies demonstrated preserved sensory nerve action potentials, low compound motor action potential amplitudes, and active and chronic denervation in the left deltoid, triceps, first dorsal interosseous, and right medial gastrocnemius, with complex repetitive discharges in the mid-thoracic paraspinal muscles. Spinal MRI was unremarkable. Genetic testing identified a heterozygous *SOD1* c.122A > G (p.Glu41Gly) variant. Based on revised El Escorial criteria [3], he met criteria for probable ALS, supported by family history and pathogenic *SOD1* mutation. Riluzole was initiated; edaravone was deferred due to planned enrollment in a tofersen clinical trial. Several months later, he developed granulomatous anterior uveitis and lumbar radicular symptoms. Biopsy of hilar lymph nodes confirmed sarcoidosis, with repeat spinal imaging remaining normal. These manifestations occurred prior to initiation of tofersen and in the absence of systemic sarcoidosis treatment. Following his fifth dose of tofersen, he developed back pain, ascending numbness, urinary incontinence, and worsening weakness. Spinal MRI demonstrated multiple expansile T2-hyperintense, gadolinium-enhancing short-segment intramedullary lesions within the cervical and thoracic cord (Fig. 1), as well as a punctate enhancing lesion in the ventral inferior medulla. Cerebrospinal fluid analysis revealed lymphocytic pleocytosis and elevated protein; infectious and neoplastic causes were excluded. He was diagnosed with probable neurosarcoidosis based on Zajicek’s criteria [4]. He improved with corticosteroids, infliximab, and mycophenolate. Subsequent clinical worsening due to anti-infliximab antibodies and suspected tofersen-associated inflammatory exacerbation prompted discontinuation of both agents and initiation of adalimumab, resulting in sustained clinical and radiologic stability. He declined tofersen rechallenge. At last follow-up, he remained wheelchair-bound with preserved distal upper limb function, required nocturnal non-invasive ventilation, and had no recurrence of neurosarcoidosis over five years.

## Discussion

The coexistence of amyotrophic lateral sclerosis (ALS) and systemic sarcoidosis has been described in several reports in the literature; in our review, we identified 12 [5–9] previously reported cases summarized in Table 1. The patients ranged in age from 29 to 63 years. There were 7 females and 5 males. The most common type of sarcoidosis involved was pulmonary, followed by muscle, with neurosarcoidosis reported in only one case. Limb-onset ALS was the predominant presentation in the majority of cases. None of the cases included a family history of ALS. Systemic sarcoidosis responded variably to treatment with corticosteroids or steroid-sparing immunosuppressants. However, ALS progression was relentless in all cases despite these interventions. Of the six patients reported in detail, two died, one required ventilatory support, and the remaining experienced continued disease progression with worsening symptoms [5–9].

**Table 1:** Summary of literature review of coexisting sarcoidosis and amyotrophic lateral sclerosis (ALS).

Case	Age/Gender	Type of sarcoidosis	Biopsy	Autopsy	Treatment	ALS Features	EMG	MRI/CSF	Outcome
1	56/F	Pulmonary, hilar lymphadenopathy	Hilar LNDs: non-caseating granulomas	No autopsy	Prednisolone 20 mg, riluzole	Limb onset.	2 regions showed denervation and reinnervation	Normal CSF and brain/spinal cord MRI	Severe muscle weakness, progression of ALS
2	63/F	Pulmonary, muscle sarcoidosis	Gastrocnemius: non-caseating granulomas	Axonal spheroid in ant horn cells, bunina bodies in residual neurons, granulomas in lymph nodes, lungs	Prednisolone 40 mg, IV methylprednisolone, plasmapheresis.	Limb-onset.	Not done	Normal CSF and spinal MRI	Death from bulbar palsy
3	39/M	Pulmonary, mediastinal lymph nodes	Hilar LNDs: non-caseating granulomas	No Autopsy	Prednisolone 30 mg	Bulbar onset	2 regions showed denervation and reinnervation	Normal brain MRI (C+) and CSF	Improved resp symptoms and transient improvement of bulbar symptoms
4	61/F	Neurosarcoidosis, systemic sarcoidosis	No biopsy	Non caseating granuloma seen lungs/LNDs/motor neurons intracytoplasmic Bunina bodies and skein-like inclusions which expressed ubiquitin and TDP-43, strongly supporting the diagnosis of ALS.	Riluzole, Baclofen	Limb-onset	3 regions showed denervation and reinnervation	Normal brain/cervical MRI and CSF	Death from aspiration pneumonia
5	41/M	Lung sarcoidosis	Mediastinal LNDs: non-caseating granulomas	No autopsy	Prednisone 1 mg/kg/day, azathioprine150 mg/day	Limb onset	3 regions showing denervation and active reinnervation.	Brain/cervical MRI showed pyramidal tract signal normal CSF	Worsened neurological symptoms
6	29/M	Pulmonary, mediastinal lymphadenopathy, muscle sarcoidosis	Lung: non-caseating granulomas, Muscle: sarcoid granulomas	No autopsy	1 g of IV Methylprednisolone, prednisone, infliximab	Limb onset	Diffuse Denervation, reinnervation consistent with MND *no regions reported	Normal brain/cervical MRI and CSF	Ventilator dependency, no improvement

M = male; F = Female; LNDs = lymphadenopathy; CSF = Cerebrospinal Fluid; IV = intravenous.

Post-mortem pathological examination in two cases revealed features of ALS and systemic sarcoidosis [7, 8]. However, neurological involvement was observed in only one case [8]. Sarcoid granulomas were found to primarily target motor regions, including the anterior horns, brainstem motor nuclei, and motor cortex, along with hallmark ALS findings such as neuronal loss observed in the ventral horns, with the most severe involvement at the cervical levels. The few surviving motor neurons exhibited intracytoplasmic Bunina bodies and skein-like inclusions positive for ubiquitin and TDP-43 [8]. These findings support true pathological coexistence rather than diagnostic mimicry and suggest a potential interaction between inflammatory and neurodegenerative processes.

Neurosarcoidosis in the context of a genetically confirmed SOD1 mutation associated with ALS has not been previously reported. We propose that the previously reported case of neurosarcoidosis and ALS likely represents non-SOD1 sporadic ALS (sALS), given the presence of TDP-43 pathology and the absence of SOD1 accumulation in motor neurons. Of note, there are cases of reported TDP-43 alongside SOD1 in genetically confirmed SOD1-ALS [9].

Tofersen, an antisense oligonucleotide (ASO) therapy that reduces mutant SOD1 production, appeared to worsen our patient’s sarcoid myelitis and radiculitis. In clinical trials, inflammatory neurological adverse events—including aseptic meningitis, radiculopathy, increased intracranial pressure, and myelitis occurred in about 7% of participants [10]. In our patient, active systemic sarcoidosis likely created an inflammatory milieu that increased susceptibility to such complications. As sarcoidosis involves granulomatous immune activation within the CNS, intrathecal therapy may amplify local inflammatory responses [2]. This overlap complicates interpretation of therapeutic efficacy, as functional decline cannot be attributed solely to motor neuron degeneration. In our case, discontinuation of tofersen and escalation of immunosuppressive therapy resulted in stabilization of the inflammatory disease without clear modification of the ALS course. These observations highlight the need for caution when interpreting the clinical effects of tofersen in patients with concurrent neuroinflammatory disorders [10].

The treatment of such cases requires a careful balance to address the inflammatory aspects of sarcoidosis while not exacerbating the neurodegenerative processes of ALS. Further research is needed to understand the mechanisms linking these conditions and to develop optimized management strategies.
